# The role of p21-activated kinases in hepatocellular carcinoma metastasis

**DOI:** 10.1186/1750-2187-9-7

**Published:** 2014-08-01

**Authors:** Edith Yuk Ting Tse, Yick Pang Ching

**Affiliations:** 1 Department of Anatomy, Li Ka Shing Faculty of Medicine, The University of Hong Kong, Hong Kong, China; 2 State Key Laboratory for Liver Research, The University of Hong Kong, Hong Kong, China

**Keywords:** Hepatocellular carcinoma, p21-activated kinases, Cdc42, Rac1

## Abstract

The p21-activated kinases (PAKs) are downstream effectors of the Rho family small GTPases as well as a wide variety of mitogenic factors and have been implicated in cancer formation, development and metastasis. PAKs phosphorylate a wide spectrum of substrates to mediate extracellular signals and regulate cytoskeletal remodeling, cell motility and survival. In this review, we aim to summarize the findings regarding the oncogenic role and the underlying mechanisms of PAKs signaling in various cancers, and in particular highlight the prime importance of PAKs in hepatocellular carcinoma (HCC) progression and metastasis. Recent studies exploring the potential therapeutic application of PAK inhibitors will also be discussed.

## Introduction

Liver cancer (hepatocellular carcinoma, HCC) is the fifth most common cancer in the world and particularly prevalent in Eastern Asia including China, Taiwan, Korea and Japan [1,2]. Hepatocarcinogenesis is a multistep process, involving the chronic infections of hepatitis B virus (HBV) and hepatitis c virus (HCV), liver cirrhosis, aflatoxin B1 intake, alcohol abuse and the accumulations of genetic and epigenetic aberrations, resulting in the dysregulation of cellular events that perpetuate the hepatocarcinogenesis process [3]. HCC is often diagnosed at advanced stage due to the lack of observable symptoms in early stage HCC. Treatment options of advanced metastasized HCC are limited as radical surgery, radiotherapy and liver transplantation are effective mainly for localized primary tumors [4]. As a result, the prognosis is extremely unsatisfactory with high incidences of postoperative recurrence and metastasis, leading to poor survival rate of HCC patients [5]. Molecular targeted therapy is therefore envisioned as potential treatment regimen to improve the survival benefits of HCC patients. Clarifying the molecular mechanisms involved in HCC development and metastasis would definitely warrant the advancement of molecular-targeted therapies. Recently, PAKs, downstream effectors of the Rho family small GTPases as well as a wide variety of mitogenic factors, have been implicated in HCC progression and metastasis. In this review, we will highlight the expression and signaling pathways of PAKs in human cancers, in particular its functional role in hepatocarcinogenesis, furthermore, the potential therapeutic application of PAK1 inhibitor will be discussed.

## Review

### PAKs signaling

p21-activated kinases (PAKs) are best characterized as the downstream effectors of the Rho family small GTPases Cdc42 and Rac1, which control the formation of filopodia and lamellipodia respectively [6]. Cdc42 and Rac1 are well established key regulators of cell migration and invasion processes involved in cancer metastasis [7], therefore it is not surprising for PAKs to be implicated in mediating extracellular signals and regulating cell motility and morphology, cytoskeletal remodeling, cell proliferation and apoptosis, thus contributing to cancer formation, development and metastasis [8].

**Figure 1:**
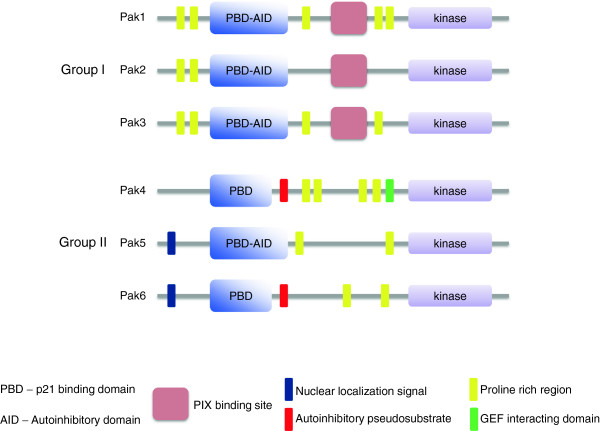
**Domain structure of PAKs.** A conserved N-terminal p21/GTPase domain (PBD) and a C-terminal serine/threonine kinase domain are shared by all family members of PAK. An autoinhibition mechanism is found available in all PAKs. Binding of activated Cdc42 or Rac1 to the PBD releases group I PAKs from the auto-inhibitory (AID) domain and activates the kinases. AID is also present in PAK5, whereas an autoinhibitory psuedosubstrate has been indentified in PAK4 and PAK6.

The PAK family members are categorized into two groups based on their structural and biochemical discrepancies: PAK1, PAK2 and PAK3 belong to group I, whereas PAK4, PAK5 and PAK6 belong to group II. The domain structure of the group I and II PAKs is depicted in Figure 1. In general, all members of group I PAKs are comprised of two Src homology 3 (SH-3)-binding motifs and a distinctive p21/GTPase binding domain (PBD) overlapped with an auto-inhibitory domain (AID) at the N-terminal region and a conserved non-classical SH3-binding site for the binding of guanine-nucleotide-exchange factor PAK-interacting exchange factor (PIX) [9], whereas the kinase domain can be found at the C-terminal. Contrastingly, only the PBD and kinase domains can be found present in the group II PAKs, lacking the AID (except PAK5 [10]), SH3- and PIX-binding domains (Figure 1). Recently, studies have revealed the presence of an autoinhibitory pseudosubstrate in PAK4 and PAK6 primary sequences, explaining the mechanism that underlies the constitutive autoinhibition of the kinases [11,12]. Both group I and II PAKs have highly conserved proline-rich domains, the kinase domains of group I PAKs share a high percentage homology of 92-95%, while the percentage identity of group II PAKs is around 80% [13].

Despite the similarity in domain structure and sequence of PAKs, the two groups actually have a pronounced difference in terms of regulatory mechanisms, substrate recognitions and cellular functions. For group I PAKs, binding of GTP-bound active Cdc42 and Rac to the PBD domain releases the kinase from the inhibition of AID, as a result, the kinase activity is enhanced [14]. Notably, binding of adaptor protein Nck and Grb2 to the two SH3-binding motifs [15,16] suggest the possibility for the recruitment of group I PAKs to the plasma membrane. Interestingly, adhesion to extracellular matrix has been found to be essential for Rac to activate PAK1 [17]. Likewise, localization of the PAK1 to the plasma membrane is indispensible for kinase activation, and the adaptor protein Nck is believed to mediate the membrane targeting [18]. In contrast, binding of activated Cdc42 or Rac to group II PAKs is not necessary for kinase activation, instead such association induces the translocation of group II PAKs to various cellular compartments [19-21]. Binding of activated Cdc42 to PAK4 targets the kinase to the Golgi apparatus, leading to actin polymerization [19,22]. Co-expression of PAK4 and constitutively active Cdc42 dramatically prolongs filopodia formation; such observation might be ascribed to the ability of PAK4 to stabilize the actin-myosin filament assembly via LIM domain kinase (LIMK) phosphorylation [23]. For PAK5, association with activated Cdc42 has no effect on the shuttling of the kinase between mitochondria and the nucleus, in which PAK5 inhibits apoptosis via BCL2-associated death promoter (BAD) phosphorylation [24-26]. Lastly, PAK6 is characterized by its distinctive FXXMF motif that interacts directly with the androgen receptor (AR) ligand-binding domain (LBD) [27].

**Figure 2:**
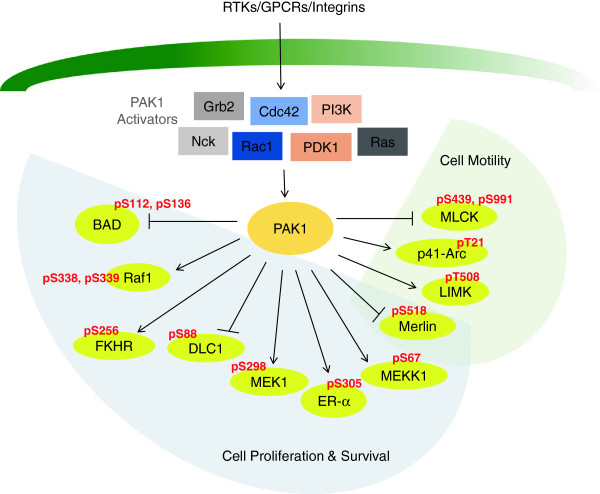
**Signaling pathways of PAK1.** The well-characterized upstream activators of PAK1 include the G protein-coupled receptors mediated Rho GTPases Cdc42 and Rac1, receptor tyrosine kinases mediated growth factor receptor-bound protein 2 (Grb2) and non-catalytic region of tyrosine kinase adaptor protein 1 (Nck), intergrins, serine/threonine kinases phosphatidylinositol 3 kinase (PI3K) and pyruvate dehydrogenase kinase isozyme 1 (PDK1) and Ras. A wide spectrum of substrates phosphorylated directly by PAK1 has been identified. Inhibition of of BAD, dynein light chain 1 (DLC1) and Merlin by PAK1 orchestrate to promote cancer cell survival. Likewise, activation of Raf1, forkhead in rhabdomyosarcoma (FKHR), MEK1, MEKK1 and estrogen receptor-α (ER-α) by PAK1 phosphorylation empower cancer cell to survive. PAK1 also phosphorylates substrates that regulate cytoskeletal dynamics, including myosin light-chain kinase (MLCK), LIM domain kinase (LIMK), p41-Arc and Merlin, cell motility is enhanced as a result.

In general, group I PAKs are known to phosphorylate substrates that play a role in cytoskeleton remodeling as well as cell survival signaling. PAK1 has been most extensively characterized among the group I PAKs and the signaling pathways of PAK1 are shown in Figure 2. The activity of PAK1 is regulated by a wide spectrum of upstream signaling molecules, including the small Rho GTPases Cdc42 and Rac, phosphoinositide-dependent kinase 1 (PDK1) [28], protein kinase A (PKA) [29], Phosphoinositide 3-Kinase (PI3K) [30] and AKT via phosphorylation and protein-protein interaction [31]. Activation of PAK1 by releasing it from the AID and the maintenance of kinase activity are resulted from the phosphorylations of Ser144, Ser199 and Ser204 and Thr423 [32,33]. Activated PAK1 then acts as an key mediator to control cell proliferation, survival, death and motility by phosphorylating corresponding downstream substrates [14]. Some of the important substrates of PAK1 and their PAK1 phosphorylation sites are depicted in Figure 2. PAK1 has been shown to play a role in cytoskeleton remodeling to increase cell motility. Activated PAK1 inhibits myosin light chain (MLC) activity by phosphorylating myosin light chain kinase (MLCK), resulting in suppression of stress fiber formation [34]. Alteration of actin dynamics by PAK1 has also been documented. PAK1 phosphorylates and activates LIMK, which then phosphorylates the actin-binding protein cofilin, resulting in inhibition of F-actin-depolymerizing activity [35]. PAK1 stimulates the actin filament nucleating protein complex Arp2/3 via phosphorylation of its p41-Arc subunit, thereby inducing cell motility [36]. PAK1 can also interact directly with various substrates to promote cell survival. Transcription factors like estrogen receptor-α (ER-α) and forkhead in rhabdomyosarcoma (FKHR) have been shown to directly interact with PAK1. PAK1 phosphorylates and induces the transactivation function of ER-α, and such activation is believed to promote hormone independence in breast cancer cells [37]. On the other hand, under the stimulation of estrogen, PAK1 phosphorylates FKHR and inhibits its nuclear translocation, repressing the ability of FKHR to activate the Fas ligand promoter that triggers apoptosis [38]. Likewise, PAK1 phosphorylates the apoptosis agonists BAD [39] and dynein light chain 1 (DLC1) [40] to inhibit their inactivating functions on the pro-survival BCL2 family members. PAK1 can inactivates the tumor suppressor Merlin via phosphorylation [41], as well as stimulating the mitogen-activated protein kinase (MAPK) cascades by phosphorylating Raf-1 [42], MEKK1 [43] as well as MEK1 [44]. The sequence of the kinase domain of group I and II PAKs is rather diverged, sharing around 50% sequence homology which suggests their difference in substrates recognitions [45]. Majority of PAK4’s substrates can also be phosphorylated by PAK1, e.g. LIMK [23], BAD [46], paxillin [47], guanine nucleotide exchange factor-H1 (GEF-H1) [48] and β-catenin [49]. PAK4 thus plays a role in cytoskeletal regulation as well as cell survival pathways.

### Dysregulation of PAKs in human cancers

The expression of PAKs in human normal and cancerous tissues is summarized in Table 1. Among the PAKs family, PAK1 is the most extensively studied member in group I PAKs; its oncogenic role and the underlying mechanisms have been investigated in a wide variety of cancers. PAK1 overexpression has been documented in various malignancies including breast [50,51], colon [52], ovarian [53], lung [54], bladder [55] and liver [56] cancers. Osada *et al.* provided the first piece of evidence that suggested the involvement of PAK1 in cellular transformation. They found that exogenous introduction of PBD domain of PAK1 could suppress fibroblast cells transformation induced by Ras and Rac [57]. It has also been documented that Ras, Raf-1 and Rac-1 induced NF-κB activation is PAK1-dependent, and PAK1 could activate NF-κB as well and stimulated the nuclear translocation of NF-κB p65 subunit [58]. In contrast, kinase dead mutant of PAK1 abolished the Ras-induced transformation in fibroblasts, demonstrating the critical role of PAK1 kinase activity in cellular transformation as well as Ras signaling [59]. Similarly, PAK1 overexpression induced lamellipodia and filopodia formations in quiescent Swiss 3T3 cells, increased cell motility via destabilization of actin stress fibres and the increase of focal adhesions turnover, suggesting its role in cancer metastasis [60,61].

**Table 1:** Expression of PAKs in human normal and cancerous tissues

		**Normal tissue**	**Overexpression in cancer**
Group I	PAK 1	Brain, muscle, spleen [6]	Bladder [55], breast [50,51], colon [52], liver [56], lung [54], kidney [85]; ovarian [53,86]
	PAK 2	Ubiquitously expressed in most tissues	Liver [74], ovarian [86]
	PAK 3	Brain [87]	
Group II	PAK 4	Ubiquitously expressed in most tissues, predominantly in prostate, testis and colon [19,22]	Colon [22], liver [76], pancreas [88]
	PAK 5	Brain [20]	Colon [89], liver [77]
	PAK 6	Brain, testis, prostate, kidney [21,45]	Prostate [90], liver [78]

In breast cancer, the activity of PAK1 is upregulated by various mechanisms. PAK1 overexpression was frequently observed and associated with breast cancer invasiveness. It was shown that PAK1 could upregulate cyclin D1 transcription in breast cancer cells thus fueling the cells with proliferative advantage [50]. Introduction of kinase-active PAK1 encouraged anchorage-independent growth and dysregulate mitotic spindles formation; the MAPK and c-Jun NH_2_-terminal kinase (JNK) activities were upregulated as well [62]. Conversely, ectopic expression of kinase dead K299R PAK1 suppressed JNK activity as well as the AP-1 promoter transcription activity, which is known to transcribe genes that involved in breast cancer invasiveness. Moreover, the phenotypic and morphogenic alterations resulted from K299R PAK1 expression included stabilization of focal adhesions and stress fibers as well as extensive cell spreading, thus reducing the motility and invasiveness of breast cancer cells [63]. Notably, PAK1 overexpression and nuclear localization were associated with Tamoxifen resistance [51], which might be explained by the ability of PAK1 to directly phosphorylate and transactivate the estrogen receptor-α (ER-α) [37]. The involvement of PAK1 in angiogenesis was revealed by the induction of vascular endothelial growth factor (VEGF) by dominant-active mutant of PAK1 in breast cancer cell [64]. Other than upregulating PAK1, hyperactivity of small GTPases Rac3 was found to augment the kinase activity of PAK1 in breast cancer [65]. Taken together, these findings provided compelling evidences that PAK1 is critical in breast cancer progression and metastasis.

The oncogenic role of PAK1 has also been studied in other cancers. In ovarian cancer, expression levels of PAK1, PAK2 and their phosphorylated forms were upregulated in both cell lines and clinical samples. In particular, PAK1 and nuclear phospho-PAK1 were associated with advanced tumor grade and poor survival rate. In search of the functional effect of PAK1 in ovarian cancer, knockdown of PAK1 markedly inhibited cell motility and invasiveness, accompanied by suppressed p38 activity and VEGF expression. Increased PAK1 expression was also found in colorectal cancer progression and significantly associated with lymph node metastases [52]. In 25% of squamous NSCLC tumors, PAK1 was found to localize in nucleus and its expression was associated with the accumulation of myeloid cell leukemia-1 (Mcl-1), which is an anti-apoptotic BCL2 family member protein. Knockdown of PAK1 could significantly suppress *in vitro* and *in vivo* tumor cell growth [54]. The anti-apoptotic effect of PAK1 is ascribed to its ability to phosphorylate BAD, a pro-apoptotic protein that regulates the intrinsic cell death machinery, causing BAD to dissociate from BCL2 and associate with 14-3-3tau, cell survival is promoted as a result, thus empowering tumor cells the advantage to proliferate [39].

PAK4 from group II PAKs is also implicated in oncogenic transformation. PAK4 is overexpressed in various cancer cell lines and Ras related tumors [22,66,67]. PAK4 activity is essential to support the anchorage-independent growth of cancer cells induced by Ras. Constitutively active PAK4 mutant S474E was found to transform NIH3T3 cells. Conversely, kinase-inactive PAK4 K350A and K351A abrogated the Ras-driven oncogenic transformation and suppressed the anchorage-independent growth of colon cancer cells [22]. Mechanistically, PAK4 was shown to exhibit anti-apopototic function by inhibiting the pro-apoptotic protein BAD as well as the caspases activity [46].

### The oncogenic functions of PAKs in HCC

The oncogenic role of PAKs has been shown in HCC, particularly in enhancing HCC progression and metastasis by promoting the motility and invasiveness of HCC cell. PAK1 overexpression was frequently observed in HCC and significantly associated with more aggressive and metastatic tumor phenotypes as well as advanced tumor stage. Overexpression of PAK1 mRNA was found in 75% of tumorous tissues and associated significantly with the presence of venous invasion, poor cellular differentiation, advanced tumor stages as well as shorter disease-free survival. Interestingly, PAK1 protein was expressed at the membrane of non-tumorous hepatocytes, while it was detected in both the membrane and cytoplasm of HCC cells. Ectopic expression of PAK1 enhanced HCC cell motility by suppressing stress fiber and focal adhesion complex formation, suggesting the role of PAK1 in HCC metastasis. Mechanistically, PAK1 was found to activate JNK and subsequently phosphorylate paxillin at Ser178 [56]. Notably, in a diethylnitrosamine (DEN)-induced liver carcinogenesis animal model, PAK1 overexpression was observed along HCC progression, together with the upregulation of cyclin D1 and activation of ERK1/2, p38 as well as JNK1/2 kinases [68], strongly suggesting its contribution in HCC development and progression. In agreement with the oncogenic effect of PAK1 in HCC, treatment of IPA-3 (2,2-dihydroxy-1,1-dinaphthyladisuifide), a highly selective non-ATP-competitive allosteric PAK1 inhibitor, could markedly suppress the *in vitro* and *in vivo* growth of HCC via induction of apoptosis and inhibition of NF-κB activation [69]. PAK1 activation has consistently been shown to promote anoikis resistance in hepatoma cells. Activation of VEGFR2/PAK1 and EGFR/PAK1 signaling, by the expression of Klotho and N-acetylglucosaminyltransferase V respectively, inhibited anoikis and such effect could be reversed by treatment of PAK1 inhibitor IPA-3 [70,71]. HBV infection is a well-established risk factor for HCC development; in fact, chronic HBV infection is associated with majority of HCC cases. Xu *et al.* has shown that PAK1 expression could be induced by the introduction of hepatitis B virus X protein (HBx) to hepatoma cells. Expression of HBx favored anchorage-independent growth as well as anoikis resistance via the upregulation of mitochondrial BCL2 and PAK1 levels. Knockdown of PAK1 suppressed *in vivo* tumor growth of HBx expressing cells as well as their resistance to anoikis. Indeed, clincopathological analyses revealed a significant correlation of PAK1 with HBV infection, poor prognosis and portal vein tumor thrombosis [72]. Interestingly, PAK1 activation by the mammalian target of rapamycin (mTOR)/p70 S6 kinase rather resulted in the suppression of HCV replication [73]. Sato *et al.* demonstrated the central role of PAK2 in mediating transforming growth factor-beta (TGF-β) signaling. TGF-β treatment induced hepatoma cell migration via phosphorylation of AKT and PAK2, while treatment of AKT inhibitor inhibited PAK2 phosphorylation, indicating PAK2 as a downstream mediator of TGF-β and AKT signaling. Inversely, downregulation of PAK2 expression impeded cell motility induced by TGF-β treatment by increasing focal adhesions formation. Clinically, the expression of phosphorylated PAK2 was significantly correlated with HCC progression and metastasis [74].

Similar to group I PAKs, group II PAKs play a definitive role in HCC growth and metastasis as well. A study of miRNomes in HCC identified a suppression of miR-199a/b-3p in HCC as compared to normal liver, and such diminution was significantly correlated to poor survival rate. Interestingly, miR-199a/b-3p actually targeted PAK4 and subsequently inhibited the PAK4/Raf/MEK/ERK signaling pathway, leading to HCC growth suppression [75]. Mak *et al.* identified CDK5RAP3 as a novel binding partner of PAK4 and found that CDK5RAP3 was frequently overexpressed in HCC with a significant correlation with PAK4 expression. Mechanistically, binding of CDK5RAP3 to PAK4 activated the kinase and subsequently enhanced the invasiveness of HCC cells [76]. Overexpression of PAK5 mRNA was also noted in 83% (25/30) of HCC samples and numerous HCC cell lines, *in vitro* studies showed that knockdown of PAK5 markedly inhibited cell proliferation and induced G1 cell cycle arrest. Downregulation of PAK5 could also suppress the *in vivo* tumor formation, accompanied by diminution of cyclin D1 and β-catenin [77]. Overexpression of PAK6 protein was found in 50% (60/121) HCC samples whereas the non-tumorous tissues showed no or low PAK6 expression. PAK6 expression was significantly correlated with high-grade tumors as well as the formation of multiple tumor nodules and notably with poor overall survival, serving as an independent prognostic marker for HCC [78]. Taken together, these studies underscore the prime importance of PAKs in HCC progression and metastasis, intervening PAKs signaling thus holds the promise of more effective therapeutic approach for HCC.

### Potential therapeutic application of PAK inhibitors

Hyperactivation of PAK1 has been reported in numerous cancers, thus making PAK1 appealing as pharmacological target for gene therapy. Several small chemical molecules like FRAX-597 and FL172 have been reported to inhibit PAK1 activity by targeting the ATP-binding or kinase catalytic site, yet their specificity towards PAK1 is unsatisfactory [79,80]. Peptides derived from the auto-inhibitory domain of PAK1 have been used to block the kinase activity, however the efficacy of such alternative approach remains to be evaluated [81]. Recently, Deacon *et al.* has reported the identification of a specific allosteric inhibitor of PAK1, called IPA-3 [82], which was suggested to bind and stabilize the auto-inhibitory domain of PAK1, resulting in inhibition of PAK1 activation. However, such suggestion needs to be proved by further study of the structures of IPA-3 and PAK1 complex, moreover, the fact that IPA-3 inhibits PAK1 activity at micro-molar level greatly impedes its application as a therapeutic drug. Our recent study has shown that IPA-3 suppressed the growth of HCC tumor in nude mouse, blocked the activation of NF-κB via PAK1 inhibition and attenuated the survival signal of HCC cells [69]. Overexpression of PAK1 is frequently observed in metastatic HCC, thus making IPA-3 an attractive therapeutic agent in treating advanced liver cancer.

As for group II PAK inhibitors, LCH-7749944 and PF-3758309, which are small-molecule inhibitors of PAK4, have been examined in recent years [83,84]. LCH-7749944 was found to suppress gastric cancer cell proliferation and invasion via inhibition of the PAK4/c-Src/EGFR/cyclin D1, PAK4/LIMK1/cofilin and PAK4/MEK-1/ERK1/2/MMP2 pathways [84]. The pyrrolopyrazole inhibitor PF-3758309, which was isolated from a high-throughput screening of molecules that inhibit both Group I and II PAKs activity, has been shown to suppress *in vivo* cancer cell growth [83]. However, due to its ATP-competitive nature, it is likely for PF-3758309 to exhibit strong toxicity on non-tumorous cells. To conclude, despite the effectiveness of PAKs inhibitors to suppress cancer cell growth and invasion, further investigations and modifications have to be done before applying the inhibitors clinically.

## Concluding remarks

Dysregulation of PAKs signaling plays an unequivocal role in the development of numerous cancers; its role in HCC metastasis has been clearly shown and supported by evidences from various lines of studies. As a mediator, PAKs converge the extracellular signals, interact and phosphorylate pools of intracellular substrates, resulting in the promotion of cancer cell survival and invasiveness. It is enormously important to target PAKs due to the central role these kinases play in cancer progression. Despite the fact that PAK1 and PAK4 inhibitors hold promise for anticancer therapy, achieving this clinically remains a continuous challenge to researcher.

## Abbreviations

HCC: Hepatocellular carcinoma; HBV: Hepatitus B virus; HCV: Hepatitus C virus; PAKs: p21-activated kinases; SH3: Src homology 3; PBD: p21-binding domain; AID: Auto-inhibitory domain; PIX: Pak-interacting exchange factor; LIMK: LIM domain kinase; BAD: BCL2-associated death promoter; AR: Androgen receptor; LBD: Ligand-binding domain; PDK1: Phosphoinositide-dependent kinase 1; PKA: Protein kinase A; PI3K: Phosphoinositide 3-kinase; MLC: Myosin light chain; MLCK: Myosin light chain kinase; FKHR: Forkhead in rhabdomyosarcoma; DLC1: Dynein light chain 1; MAPK: Mitogen-activated protein kinase; GEF-H1: Guanine nucleotide exchange factor-H1; JNK: c-Jun NH_2_-terminal kinase; ER-α: Estrogen receptor-α; VEGF: Vascular endothelial growth factor; Mcl-1: Myeloid cell leukemia-1; DEN: Diethylnitrosamine; HBx: Hepatitis B virus X protein; mTOR: Mammalian target of rapamycin; TGF-β: Transforming growth factor-beta.

## Competing interests

The authors declare that they have no competing interests.

## Authors’ contributions

Both authors reviewed the literature in this review, drafted, prepared approved the manuscript.
